# Quality of life in patients with allergic and immunologic skin diseases: in the eye of the beholder

**DOI:** 10.1186/s12948-021-00165-6

**Published:** 2021-12-20

**Authors:** Ester Di Agosta, Lorenzo Salvati, Monica Corazza, Ilaria Baiardini, Francesca Ambrogio, Luisa Angileri, Elettra Antonelli, Federica Belluzzo, Domenico Bonamonte, Laura Bonzano, Raffaele Brancaccio, Paolo Custurone, Aurora De Marco, Aikaterini Detoraki, Adriana Di Guida, Elisabetta Di Leo, Marta Fantò, Filippo Fassio, Silvia Mariel Ferrucci, Caterina Foti, Rosella Gallo, Alessia Gatta, Fabrizio Guarneri, Lucia Guidolin, Katharina Hansel, Donatella Lamacchia, Carla Lombardo, Paola Lucia Minciullo, Maddalena Napolitano, Alessandro Pannofino, Andrea Paravisi, Roberta Parente, Maria Passante, Cataldo Patruno, Diego Peroni, Cristina Quecchia, Natale Schettini, Giuseppe Spadaro, Luca Stingeni, Daniele Tarrini, Marta Tramontana, Eustachio Nettis, Oliviero Rossi

**Affiliations:** 1 Department of Experimental and Clinical Medicine, University of Florence, Firenze, Italy; 2 Section of Dermatology and Infectious Diseases, Department of Medical Sciences, University of Ferrara, Ferrara, Italy; 3 Respiratory Unit for Continuity of Care, IRCCS Ospedale Policlinico San Martino, Department of Internal Medicine (DiMI), University of Genoa, Genova, Italy; 4 Personalized Medicine, Asthma and Allergy, IRCCS Humanitas Research Hospital, Rozzano, Milano, Italy; 5 Department of Biomedical Science and Human Oncology, Section of Dermatology, University of Bari, Bari, Italy; 6 Dermatology Unit, Fondazione IRCCS Ca’ Granda Ospedale Maggiore Policlinico, Milano, Italy; 7 Section of Dermatology, Department of Medicine and Surgery, University of Perugia, Perugia, Italy; 8 ASP Trapani, Trapani, Italy; 9 Department of Biomedical Sciences and Human Oncology, Section of Dermatology, University of Bari, Bari, Italy; 10 Dermatology Unit, Azienda USL – IRCCS di Reggio Emilia, Reggio Emilia, Italy; 11 Division of Allergy and Clinical Immunology, “Santa Maria Della Speranza” Hospital, Salerno, Italy; 12 Section of Dermatology, Department of Clinical and Experimental Medicine, University of Messina, Messina, Italy; 13 Department of Internal Medicine, Clinical Immunology, Clinical Pathology and Infectious Disease, Azienda Ospedaliera Universitaria Federico II, Napoli, Italy; 14 Section of Dermatology, Department of Clinical Medicine and Surgery, University of Naples Federico II, Napoli, Italy; 15 Section of Allergy and Clinical Immunology, Unit of Internal Medicine-“F. Miulli” Hospital, Acquaviva Delle Fonti, Bari, Italy; 16 Unit of Allergology, Department of Dermatology, Policlinico Umberto I, Hospital-University Sapienza of Rome, Roma, Italy; 17 Allergy and Clinical Immunology Unit, San Giovanni di Dio Hospital, Firenze, Italy; 18 Section of Dermatology - Department of Health Sciences, University of Genoa, Ospedale Policlinico San Martino - IRCCS, Genova, Italy; 19 Department of Medicine and Science of Ageing, G. d’Annunzio University, Chieti, Italy; 20 Allergy Unit, Department of Medicine, University of Verona, Verona, Italy; 21 Allergy Unit, Villa Igea Hospital. A.P.S.S. Trento, Trento, Italy; 22 Department of Clinical and Experimental Medicine, School and Division of Allergy and Clinical Immunology, University Hospital ‘G. Martino’, Messina, Italy; 23 Department of Medicine and Health Science “V. Tiberio”, University of Molise, Campobasso, Italy; 24 Allergology and Immunology Clinic, Operative Unit of Medicine, Policoro Hospital, Policoro, Matera, Italy; 25 Section of Dermatology, Department of Health Sciences, University of Genoa, Ospedale Policlinico San Martino - IRCCS, Genova, Italy; 26 Department of Medicine, Division of Allergy and Clinical Immunology, University of Salerno, Salerno, Italy; 27 Department of Health Sciences, University Magna Graecia of Catanzaro, Catanzaro, Italy; 28 Section of Pediatrics, Department of Clinical and Experimental Medicine, University of Pisa, Pisa, Italy; 29 Io e l’Asma Center, Children’s Hospital, ASST Spedali Civili, Brescia, Italy; 30 Department of Translational Medical Sciences and Center for Basic and Clinical Immunology Research (CISI), University of Naples Federico II, Napoli, Italy; 31 Allergy and Clinical Immunology Unit, Department of Clinical Medicine, Mauriziano Umberto I Hospital, University of Turin, Torino, Italy; 32 Department of Emergency and Organ Transplantation, School and Chair of Allergology and Clinical Immunology, University of Bari - Aldo Moro, Bari, Italy; 33 Immunoallergology Unit, SOD Immunoallergologia, Azienda Ospedaliero-Universitaria Careggi, Firenze, Italy

**Keywords:** Quality of life, Atopic dermatitis, Allergic contact dermatitis, Hereditary angioedema, Cutaneous mastocytosis, Urticaria, Skin, Allergy

## Abstract

Allergic and immunologic skin diseases negatively impact the quality of life (QoL) of affected patients with detrimental consequences. Nonetheless, in everyday clinical practice the evaluation of QoL is often overlooked. Considering the increasing prevalence of atopic dermatitis, allergic contact dermatitis, hereditary angioedema, cutaneous mastocytosis, and urticaria, it is essential to determine the effects of allergic and immunologic skin diseases on QoL. A joint meeting (GET TOGETHER 2021) of the Italian Society of Allergology, Asthma and Clinical Immunology (SIAAIC) and the Italian Society of Allergological, Occupational and Environmental Dermatology (SIDAPA) aimed to summarize the features of the main QoL tools used in these diseases and to describe the extent of QoL impairment as well as the impact of treatments on QoL, particularly biologic therapies. The assessment of QoL in patients with allergic and immunologic skin diseases relies on generic, organ-specific and disease-specific questionnaires. While generic and organ-specific questionnaires allow comparison between different diseases, disease-specific questionnaires are designed and validated for specific cohorts: the QoL Index for Atopic Dermatitis (QoLIAD) and the Childhood Atopic Dermatitis Impact Scale (CADIS) in atopic dermatitis, the ACD-11 in allergic contact dermatitis, the Angioedema QoL Questionnaire (AE-QoL) and the Hereditary Angioedema QoL questionnaire (HAE-QoL) in hereditary angioedema, the Mastocytosis QoL Questionnaires (MCQoL e MQLQ) in cutaneous mastocytosis, and the Chronic Urticaria QoL questionnaire (CU-Q2oL) in urticaria. Among the many factors that variably contribute to QoL impairment, pruritus can represent the leading cause of patient discomfort. Biologic therapies significantly ameliorate QoL in atopic dermatitis, hereditary angioedema, mastocytosis and chronic urticaria. In general, adequate management strategies are essential for improving QoL in patients with allergic and immunologic skin diseases.

## Background

*“The value of experience is not in seeing much, but in seeing wisely” Sir William Osler [1849–1919]*

Quality of life (QoL) of patients with allergic and immunologic skin diseases is often a neglected issue in clinical practice [1]. The GET TOGETHER 2021 meeting, organized by SIAAIC (Società Italiana di Allergologia, Asma ed Immunologia Clinica) and SIDAPA (Società Italiana di Dermatologia Allergologica, Professionale e Ambientale) was a virtual meeting held by specialists in allergic and immunologic skin diseases in Italy between May and June 2021 with the primary aim to discuss and review the current knowledge on the QoL of patients with atopic dermatitis, allergic contact dermatitis, hereditary angioedema, cutaneous mastocytosis, and urticaria. Considering the increasing prevalence of allergic and immunologic skin disorders, it becomes fundamental to evaluate their impact on the QoL of affected patients, as well as the effect of current therapies on QoL.

## Main text

### Generic Questionnaires: assessing QoL in patients with allergic and immunologic skin diseases

*Generic questionnaires* are designed to assess health related quality of life (HRQoL) in wide populations with or without chronic conditions and they allow comparing different diseases [2, 3]. The most used are the Short Form 36 Health Survey (SF-36), the EuroQol-5D (EQ-5D) and the Nottingham Health Profile (NHP).

- The *Short Form 36 Health Survey (SF-36)* is a self-administered, multidimensional, well validated and widely used generic instrument [3]. It consists of 36 items relating to eight dimensions (physical function, role-physical function, bodily pain, general health perceptions, vitality, social functioning, role-emotional function and mental health), one physical component score and one mental component score. The eight-dimension score range is 0–100, with higher scores indicating higher levels of function and⁄or better health. The SF-36 also includes a general health rating item, which inquires about the change in general health over the last year [4].
- The *EuroQol-5D (EQ-5D)* is a generic and synthetic measure of the HRQoL. It essentially consists of two parts: the EQ-5D descriptive system and the EQ visual analogue scale (EQ VAS). The EQ-5D descriptive system comprises five dimensions: mobility, self-care, usual activities, pain/discomfort and anxiety/depression [5]. Each dimension is analyzed according to different graduated levels: EQ-5D-3L (no, some, or extreme problems) and EQ-5D-5L (no, slight, moderate, severe, or extreme problems). The EQ VAS records the patient’s self-rated health on a vertical VAS where the endpoints are labelled as “best imaginable health state” and “worst imaginable health state” [5].
- The *Nottingham Health Profile (NHP)* was developed for measuring the impact of disease on patients and the assessment of changes in health status over time. It provides a brief indication of a patient’s perceived emotional, social, and physical health and is intended for use in the general population. The NHP is composed of two parts that can be used together or separately, with the first part frequently used on its own. The domains covered in the first part are related to health status, while the second part addresses the impact of disease on daily life [6].

*Organ-specific questionnaires* investigate one system, differently from generic or disease-specific questionnaires. Their advantage is the application for assessment of different dermatological diseases; however some skin diseases also involve other organs not considered in these skin-specific tools [2]. In dermatology, commonly used organ-specific instruments include the Dermatology Life Quality Index (DLQI), the Skindex-29 and the Dermatology-Specific Quality of Life (DSQL).

- The *Dermatology Life Quality Index (DLQI)* and the corresponding *Children’s Dermatology Life Quality Index (CDLQI)* used for patients < 16 years, are self-administered questionnaires consisting of 10 questions concerning the impact of skin diseases on different aspects of patient's QoL over the last week [7]. The DLQI items include symptoms and feelings, daily activities, leisure, work or school, personal relationships and the side effects of treatment [7].
- The *Skindex-29* was designed to measure health-related QoL in skin conditions, allowing changes over time to be detected. Its final version consists of 29 distinct questions in three categories: emotions, activities and symptoms. Subsequently, two short versions were developed (Skindex-16 and Skindex-17), originated from two different sub-categories: psycho-social and symptoms [4].
- The *Dermatology-Specific Quality of Life (DSQL)* provides valid and reliable assessments of QoL impairment associated with acne and contact dermatitis; it is used to quantify the effects of skin disease on physical discomfort and symptoms, psychological well-being, social functioning, self-care activities, performance at work or school, and self-perceptions [8, 9].

### Quality of life in patients with atopic dermatitis

Atopic dermatitis (AD) is a common inflammatory skin disease characterized by intense pruritus and recurrent eczematous lesions that affects both children and adults [10]. Itching represents the main cause of patient discomfort [11]. AD extensively interferes with sleep: patients have difficulties in falling asleep, tend to awake at night and have reduced sleep hours with consequent daytime sleepiness, reduced work or school performance and irritability [11]. The emotional and behavioral domains are also affected. In children, AD interferes with physical abilities (poor participation in sports activities), often changing relationships with peers and teachers, but the entire family is involved in terms of lower social support, higher stress, and greater difficulties in managing discipline [12, 13]. As a result, the psychological development of the child with AD can be impaired [13]. Age and gender can affect the different perception of QoL [14]. Severe AD is associated with greater QoL impairment [15]. Often in AD, adequate anti-pruritic therapy determines QoL improvement.

#### Atopic dermatitis-specific QoL tools

The impact of AD on QoL can be measured using several QoL questionnaires: generic and organ-specific instruments are frequently used. Among these, the DLQI has been recommended by the HOME initiative as the core instrument for measuring the impact of AD on the QoL of adult patients with AD [16]. The Quality of Life Index for Atopic Dermatitis (QoLIAD), is a disease-specific patient reported outcome for adult AD patients. It includes 25 questions with dichotomous answers, which makes it simple and practical to use even in clinical practice [17]. Several dermatology specific and AD-specific validated instruments measure the impact of QoL on family members of AD patients. Among these, the Childhood Atopic Dermatitis Impact Scale (CADIS) is a disease-specific QoL scale with 45 items for children with AD younger than 6 years and for their parents, exploring 5 domains: child symptoms, child activity limitations and behavior, family and social function, parent sleep, and parent emotions [13].

#### Impact of treatment on QoL in patients with atopic dermatitis

The goals of treatment are to reduce symptoms (pruritus and dermatitis), to prevent exacerbations and to minimize therapeutic risks. Standard treatment modalities for the management of these patients are centered around the use of topical anti-inflammatory preparations and moisturization of the skin, but patients with severe disease may require phototherapy or systemic treatment [18]. Based on the growing understanding of the pathomechanisms of AD, several biologics and small molecules targeting various AD-related pathways are being investigated in clinical trials [19]. Among these, dupilumab, a monoclonal antibody directed against the interleukin-4 receptor subunit α (IL-4Rα) of IL-4 and IL-13 receptors, is the only biologic therapy that is Food and Drug Administration approved for the treatment of moderate-to-severe AD in patients 6 years and older, with consistent long-term efficacy and safety trial data. In the first clinical trials with dupilumab in monotherapy (SOLO 1 and SOLO 2), reduction in pruritus, reduction in symptoms of anxiety or depression (Hospital Anxiety and Depression Scale, HADS), and improvement in DLQI from baseline were significantly greater in the dupilumab group than in the placebo group at week 16 [20]. In another study, dupilumab‐treated patients in monotherapy experienced a mean 7.2‐point improvement in DLQI score, compared with 1.6‐point improvement with placebo and a significantly greater proportion of dupilumab‐treated patients had ≥ 4‐point reduction (improvement) in DLQI score than with placebo (59·3% vs. 24·4%, *p* < 0.001) [21]. In CHRONOS trial, at 52 weeks an improvement of DLQI greater or equal to 4 points was achieved by 63% of patients treated with weekly dupilumab and topical corticosteroids and by 80% of patients treated with dupilumab every 2 weeks and topical corticosteroids, compared to 30% of patients treated with placebo and topical corticosteroids (*p* = 0.0001) [22]. Moreover, other studies showed significant reductions in pruritus (Numerical Rating Scale, NRS and scores for pain and discomfort (EQ‐5D) in adult patients with AD [21, 23]. Similarly, a recent post hoc analysis revealed that adolescents with moderate to severe AD treated with dupilumab experienced statistically significant and clinically meaningful improvements in AD signs, symptoms (including pruritus and sleep loss), and QoL at week 16 compared with the palcebo group [24]. The results of the registrative studies of dupilumab, regarding the improvement of the items of the QoL questionnaires administered to enrolled patients, were also confirmed by real life studies that were subsequently carried out on different groups of patients (elderly, adolescents) [25, 26]. AD is a complex and multifactorial disease with a significant impact on the QoL of affected patients as well as of their families. AD is not an easy condition with a straightforward, one-size-fits-all solution; however, in this new era of genetic and molecular discoveries, better tools to address this condition on an individualized basis will be available, thus further improving patient’s QoL [27].

### Quality of life in patients with allergic contact dermatitis

Contact dermatitis (CD) is an inflammatory cutaneous disease characterized by skin lesions occurring after contact with an exogenous substance. Classically, it comprises two different forms: irritant contact dermatitis (ICD) and allergic contact dermatitis (ACD) [28, 29]. The latter is a delayed hypersensitivity reaction triggered by skin contact with an allergen in previously sensitized patients [30]. In the acute phase, ACD is characterized by erythematous, edematous, and papulo-vesicular lesions, while scaling and lichenification are the typical clinical aspects of the chronic disease [28, 31]. Pruritus is the typical symptom of ACD. Despite the significant prevalence of ACD (it is estimated between 15 and 20% in the general population), the impact of this condition on QoL is poorly known and little has been published about the quantification of QoL of ACD patients [32, 33]. Assessment of the extent of QoL changes in patients with ACD is based on symptoms, feelings, function, occupation and treatment [34, 35]. Patients with ACD reported being bothered most by eczematous skin lesions, itching and the predisposition to persistence of disease [31]. The clinical presentation, especially if localized on the hands or face, often causes reduced esteem by self and others with a high risk for depression and anxiety [32, 36] The impact on the QoL is greater, the greater the severity of disease [37]. Ayala et al. demonstrated that four main aspects (itching, discomfort, difficulty in carrying out daily activities, and difficulty using hands at work) are of utmost importance in influencing the QoL of CD patients. The more these features are negatively altered, the easier a poor QoL can be predicted, particularly for females when compared to males [38]. ACD patients with chronic severe hand eczema can have an important functional impact in terms of limitation in manual skills and difficulty in carrying out the common gestures of daily life, as buttoning up, opening a bottle, and in many other activities. Considering that ACD and ICD represent about 90% of occupational skin diseases, occupational setting, particularly if hands are affected, is also frequently impaired, contributing to lengthy absences from work (“sick-leave”) and negatively impacting also in the socioeconomic area of the whole society [32]. In addition, ACD can result in “disability”, meaning the loss of ability in doing the working activity as compared before the disease onset. ACD can consequently result in reduced productivity and loss of quality of work thus generating possible conflicts in the workplace [39]. It is not infrequent that ACD patients are forced to change job or type of working activity resulting sometimes in professional disqualification and economic disadvantage [39]. The psychosocial impact of ACD on QoL is relevant both in occupational and non-occupational ACD and includes mainly anxiety, depression and sleep disturbance, but also difficulties in fulfilling personal and family responsibilities and limitations in leisure activities [35, 40]. The need for time (usually long-term) and economic investment (often charged to the patient) that must be dedicated to medical treatments impact negatively on QoL [41]. It is important to note that a timely indication of patch testing is critical to relieve patients’ suffering and reduce cost of treatment [42–44]. Patch testing significantly improves QoL, both symptoms domain and emotional impact [35, 44–46]. Even patients with negative results seem to benefit from this diagnostic test [47]. QoL improves about 6 months after diagnosis but worsens after 12 months from patch testing [33]. An annual follow-up may improve QoL thanks to patients’ education and constant evaluation of allergen exposures. ACD negatively impacts on the QoL of affected patients, however data on quantification of this significant burden are scarce. For this reason, we believe that an ACD-approach should be proposed in which the acronym ACD—in addition to the disease—denotes three actions that clinicians should play: Ask, Contribute, and Deter. Asking about health status is an essential question during visits to ACD patients, a few more questions should be asked just to complete a specific QoL questionnaire. Contributing to the current knowledge with good quality data that explore QoL of ACD patients referred not only to university hospitals but also to other hospitals and outpatient clinics, it would be possible to reach consensus on a comprehensive, validated and ACD-specific QoL tool. Finally, detering from exposure to culprit allergens means prevention which is essential to improve QoL of patients with ACD.

#### Allergic contact dermatitis-specific QoL tools

The instruments available to assess the burden of ACD on the HRQoL of patients are questionnaires that range from generic to disease-specific [37, 45, 48–52]. The most commonly used dermatology-specific QoL questionnaires in patients with ACD are the DLQI [39, 53, 54], the Skindex-29 or Skindex-16 [32, 33, 43, 55], and the DSQL [9, 56]. The DSQL had been originally validated in patients with contact dermatitis, but its use in this patient population is limited in the literature [9, 37]. Contact dermatitis-specific questionnaires have been developed and include the Contact Dermatitis-Specific Questionnaire [38], the Fragrance Quality of Life Index (FQLI) [57–59], the Quality of Life in Hand Eczema Questionnaire (QOLHEQ) [41, 60], and the ACD-11 [32, 35]. Among these tools, the ACD-11 has been specifically designed for patients with ACD [35]. Available contact dermatitis-specific questionnaires comprise different numbers of items, refer to different periods of time and have some limitations in patients with ACD, as outlined in Table 1. The evaluation of the impact of ACD on work productivity can be more precisely measured using the Work Productivity and Activity Impairment (WPAI) questionnaire and its various declinations [39, 53, 61, 62]. Considering the pediatric population with ACD, available QoL tools include the CDLQI questionnaire [63].

**Table 1:** Quality-of-life specific instruments in patients with allergic contact dermatitis

QoL specific tool	References [PMID]	Total no. items	Recall period	Indications	Limitations in patients with ACD
Contact Dermatitis-Specific Questionnaire	Ayala et al. [38] [20233546]	20	Last 6 months	Patients with contact dermatitis	Rarely used in the literature
Fragrance Quality of Life Index (FQLI)	Heisterberg et al. [57] [24600708]	13	Currently	Patients with fragrance allergy	Use is limited to fragrance-related allergic contact dermatitis
Quality of Life in Hand Eczema Questionnaire (QOLHEQ)	Ofenloch et al. [41] [24397866]	30	Last 7 days	Patients with hand eczema	Use is limited to hand involvement
ACD-11	Raffi et al. [35] [32049717]	11	Last 4 weeks	Patients with allergic contact dermatitis	Symptoms domain does not include pruritus, functioning domain does not include work impairment

#### Impact of treatment on QoL in patients with allergic contact dermatitis

The goal of treatment is the avoidance of the contact with the causative agent [29, 31]. If the culprit allergen or irritant is not found or eliminated, the dermatitis may become chronic with negative impact and repercussions throughout several aspects of the life of affected people with a high disease burden [31, 36]. Patient education about causes of contact allergy and avoidance of documented allergens and irritants represent the first line management approach. In case of mild disease, personal protective measures such as barrier creams, gloves and protective clothing, skin barrier repair cream, alcohol disinfection and addition of moisturizers (use of humectants and emollients) help to improve skin barrier. The mainstay of treatment is application of top﻿ical corticosteroids, often supplemented by moisturizing creams [64]. Topical calcineurin inhibitors (pimecrolimus, tacrolimus) and topical phosphodiesterase 4 inhibitor (crisaborole) are off-label use for CD but are known to be effective. In case of treatment failure, phototherapy could be considered. For moderate to severe localized dermatitis, topical anti-inflammatory treatment can be used as needed. Moreover, dietary avoidance and removal of airborne allergens may be of some benefit in recalcitrant forms of the disease [64]. The resistant nature of moderate to severe CD makes its management challenging. Phototherapy (narrow band UVB) or psoralen plus UVA (PUVA) can be used. Current systemic treatments include systemic corticosteroids (short term option) and other immunosuppressants (cyclosporine, methotrexate, azathioprine, mycophenolate mofetil). With advances in the understanding of the cellular and molecular pathogenesis of CD, emerging biologic therapies with licensed indications for other eczematous and immunoinflammatory skin conditions may have a place in the treatment of recalcitrant cases of ACD [65]. Infliximab, a TNF-α inhibitor, has been tried in patients with recalcitrant atopic eczema with associated CD [66, 67]. Dupilumab has been used successfully in systemic ACD to nickel and it has proven to be of value in the management of recalcitrant ACD [68, 69]. Omalizumab, an anti-IgE monoclonal antibody has been tried in recalcitrant protein CD to wheat [70]. The positive response to omalizumab in case of wheat allergy can be accounted to the fact that wheat allergy is a combination of type I and type IV hypersensitivity reactions. Anti-IgD antibodies are known to deplete B cells and modulate both Th1 and Th2 response and inhibit apparent skin inflammation when administered in allergen-challenged murine models: this finding could pave the way for anti-IgD antibodies to be tried as a therapeutic option in ACD [71]. Because ACD is an immunologically mediated disease, the role of biologics and other immunomodulators is undeniable [64, 65]. Further use and research of biologics, especially in cases of recalcitrant CD, may identify new treatment modalities for CD in the near future.

### Quality of life in patients with hereditary angioedema

Hereditary angioedema (HAE), firstly described by Osler in 1888 as “hereditary angioneurotic oedema”, is a rare genetic disease characterized by recurrent episodes of oedema of cutaneous and submucosal tissue, caused by temporary vasodilation, increased vascular permeability and flow of fluids into the extracellular space. A deficiency of the enzymatic activity of C1 esterase inhibitor (C1-INH), a protease inhibitor of the serpin superfamily, leads to an increased production of bradykinin, which is responsible for the clinical manifestations. Over 450 mutations in the C1-INH gene were reported [72], which lead to a reduced synthesis of C1-INH (HAE type I, ~ 85% of cases) or quantitatively normal synthesis of functionally abnormal C1-INH (HAE type II, ~ 15% of cases). Moreover, in 2000, a dominantly inherited disease has been described that has a similar clinical picture to hereditary angioedema with C1 inhibitor deficiency (C1-INH-HAE), but with normal C1-INH level and activity [73]. This very rare condition is associated in some cases with mutations in the gene of coagulation factor XII, while other cases are yet of unknown origin. HAE presents with attacks of swelling of the face, larynx, tongue, extremities, stomach, intestines, and genitals, has a self-limiting course, and a variable duration. It is accompanied by intense pain and deformity of the involved sites, and, in some cases, fatal risk may occur, due to severe oedema of the larynx. Prodromal symptoms and signs such as fatigue, tingling and marginate erythema may sometimes be observed [74]. The unpredictability of the attacks, as well as intensity and severity of the symptoms, make the disease particularly impacting on the QoL of affected patients and their caregivers. Attacks may be painful and debilitating, interfering with daily activities such as attending work or school and/or participating in social and family life. This entails a significant emotional distress and clinical burden on both patients and family members, causing a worsening of the QoL [75].

#### Hereditary angioedema-specific QoL tools

C1-INH-HAE experts recommend to measure HRQoL once a year, and the C1-INH-HAE guidelines of the World Allergy Organization state that HRQoL should be considered when assessing the need for prophylaxis [76, 77]. Currently, two angioedema-specific HRQoL questionnaires are available: the AE-QoL, specific for angioedema as a symptom, and the HAE-QoL, specific for adult patients with C1-INH-HAE (Table 2) [78].

**Table 2:** Quality-of-life specific instruments in patients with hereditary angioedema

QoL specific tool	References [PMID]	Total no. items	Recall period	Domains	Points
AE-Qol	Weller et al. [79] [22913638] Validated for angioedema (AE) as a symptom and not for C1-INH-HAE	17	Last 4 weeks	1. Functioning 2. Fatigue/mood 3. Fears/shame 4. Food	0–100
HAE-Qol	Prior et al. [87] [22817696]; Prior et al. [88] [26969268] Validated for hereditary angioedema (HAE)	25	Last 6 months	1. Physical functioning and health 2. Role emotional and social functioning 3. Concern about offspring 4. Treatment difficulties 5. Disease-related stigma 6. Perceived control over illness 7. Mental Health	25–135

#### AE-QoL

The AE-QoL is the first specific patient-reported outcome tool to assess QoL impairment in adult patients with any kind of recurrent angioedema [79]. Nowadays, linguistically validated versions of AE-QoL are available for several languages [80–83]. The AE-QoL includes 17 items, grouped into four dimensions and has a recall period of 4 weeks. It exhibits good levels of internal consistency, convergent and known-groups validity as well as test-retest reliability, even if patients with different angioedema conditions (chronic spontaneous urticaria, C1-INH-HAE, idiopathic angioedema) participated in its development [79]. AE-QoL scores were found to correlate well with DLQI, SF-36, and SF-12 scores, and with disease activity [80, 83]. In addition, AE-QoL is also sensitive to changes, with a Minimal Clinically Important Difference (MCID) of six points [80]. The AE-QoL total score seems to be linked to angioedema attack rates; however, at an angioedema frequency of more than four attacks per 4 weeks, the AE-QoL total score increase flattens, suggesting that the angioedema-related QoL impairment reaches a plateau when the number of attacks exceeds a critical rate [79]. AE-QoL is the recommended tool for assessing QoL in urticaria patients with angioedema in the EAACI/GA2LEN/EDF/WAO guidelines for urticaria [84–86]. As it is symptom-specific rather than disease-specific, AE-QoL does not include hereditary transmission but comprises “food”, which is not a common trigger for HAE attacks.

#### HAE-QoL

The HAE-QoL draft version was carried out in a multicenter study in Spain performed in 2012 and thereafter internationalized in 2016, in a cross-cultural adaptation with 17 participating countries [87, 88]. It consists of 25 items, grouped into seven HRQoL domains for C1-INH-HAE adult patients, with a 6-month recall period [88]. The HAE-QoL has been developed following a qualitative methodology, considering the patient-centered perspective as a key issue to evaluate when assessing HRQoL [87]. After the international pilot study, HAE-QoL showed good internal consistency and test-retest reliability, as well as a good discriminant validity in the psychometric analysis [88]. An American version, the United States HAE Association (HAEA)-QoL, based on the experiences and expectations of management of US patients, was developed in 2018 [89]. Additional validation studies and assessment of reliability to measure its ability to detect changes over time are ongoing [89].

#### Impact of treatment on QoL in patients with hereditary angioedema

Despite its efficacy, the therapy to be used in acute phases, given the high risk of potentially fatal outcomes of each attack, has no remarkable impact on the psychological aspects of this condition. The QoL of patients affected by HAE greatly depends on prophylactic therapies. Injection of C1-INH, recombinant or obtained from plasma of donors, is one of the most used options in this field. As shown in several studies, C1-INH therapy significantly improved the QoL scores, in comparison with placebo [90–92]. Lanadelumab, a monoclonal antibody acting as inhibitor of active plasma kallikrein [93], has been recently added among therapeutic strategies for long-term prophylaxis in patients aged ≥ 12 years with HAE due to C1-INH-HAE [94]. The HELP study, a randomized double-blind placebo-controlled parallel-arm phase III study, other than demonstrating the efficacy of lanadelumab in reducing HAE attacks [95], investigated the effect of this biologic on patients’ HRQoL [75]. During the treatment period, a greater proportion of lanadelumab-treated patients than placebo-treated patients achieved the favorable MCID in AE-QoL scores, and, among all domains, the largest improvement was recorded in functioning, indicating fewer restrictions in work, physical activity, leisure time and social relations. Positive results of lanadelumab on HRQoL were also confirmed in the open-label extension of the HELP study (HELP-OLE), with reduction in all domain scores, particularly in fears/shame (emotional burden) followed by functioning [96, 97]. Another, smaller study prospectively assessed QoL in 12 adult HAE patients undergoing lanadelumab therapy for six months: a significant improvement was observed in all cases, together with significant reduction in the number of attacks [98].

Although further evidence is necessary, these data demonstrate that lanadelumab can dramatically improve QoL in patients with C1-INH-HAE, overcoming some limits of existing long-term prophylactic drugs such as adverse effects [99], need for frequent intravenous administrations [76], and sometimes limited availability, due to a shortage in supply (particularly in the case of plasma derived products) [100]. Moreover, a gradual extension of intervals between lanadelumab injections may be achieved without losing therapeutic efficacy, reducing the still high costs and furthering the idea of an individualized therapy [101].

### Quality of life in patients with cutaneous mastocytosis

Mastocytosis is a rare disease characterized by the abnormal proliferation of clonal mast cells (MC) in various organs. In most cases, the disease is due to a gain-of-function mutation of the KIT gene, encoding for the most important receptor on MC surface, regulating survival and proliferation of MC. The WHO classification of mastocytosis, recently updated, identifies two groups of disorders: cutaneous mastocytosis (CM) and systemic mastocytosis (SM) [102, 103]. CM is usually diagnosed during childhood and involves only the skin, with a good prognosis and a general tendency to spontaneous resolution during puberty. On the other hand, SM is usually observed in adult patients and may involve different organs other than the skin, most frequently the bone marrow. Several variants of SM have been currently identified, ranging from Indolent Systemic Mastocytosis (ISM) with an excellent prognosis and an almost normal life expectancy, to Advanced Systemic Mastocytosis (AdvSM), with less favorable outcomes [102, 103]. The clinical picture depends either on the MC-organ infiltration or on mediator-related symptoms, including urticaria, flushing, pruritus, gastrointestinal symptoms (such as chronic diarrhea), fatigue and recurrent anaphylaxis. To measure and monitor the burden of symptoms in patients with mastocytosis, two main tools have been proposed, including the Mastocytosis Activity Score (MAS) [104] whose only limitation is the low percentage of patients with advanced disease participating to the validation study, and the Mastocytosis Symptoms Assessment Form (MSAF) also useful to monitor the effects of treatments in patients with SM (Table 3) [105]. However, although the clinical presentation of the disease is quite heterogeneous, general symptoms may deeply affect the QoL of patients, regardless of the severity of the disease and other parameters such as KIT mutation and tryptase levels [105–109]. Therefore, patients affected by all forms of mastocytosis largely share everyday difficulties and problems, starting from a generally relevant delay in the diagnosis. Jennings et al., reported indeed that the years occurring between the first symptoms and the final diagnosis ranged from less than 1 year to 50 years, with an average of 6.5 years [110]. However, a consistent delay in the diagnosis not only postpones the beginning of a correct multidisciplinary evaluation, but it also affects patients’ global QoL. A quality study in 2019 demonstrated indeed that a correct diagnosis improves patients’ QoL by giving them the opportunity of following appropriate therapies as well as of better understanding and accepting their disease [110]. Moreover, typical skin lesions are very frequent in patients with ISM and represent a relevant aesthetic problem for almost all patients, as they report not to feel comfortable in public places where people can look at them [108–110]. Skin lesions and pruritus seem indeed to be the most disabling symptoms, together with the risk for recurrent anaphylaxis [108–110]. A recent study conducted on 101 adult patients with mastocytosis shows that one third of patients felt moderately or severely impaired in QoL. Moreover, according to these Authors several factors may negatively influence the QoL, including food intolerance, osteoporosis, and the need for pharmacological treatments. Therefore, patients with anaphylaxis had a total MastoCytosis Quality of Life Questionnaire (MC-QoL) score only slightly more elevated than the one scored by patients without this manifestation, although this difference was not statistically significant. Interestingly, in this study, higher serum tryptase levels and a longer duration of symptoms were associated with higher QoL impairment [111].

**Table 3:** Quality-of-life specific instruments in patients with mastocytosis

QoL specific tool	References [PMID]	Total no. items	Recall Period	Domains	Points
MastoCytosis Quality of Life Questionnaire (MC-QoL)	Siebenhaar et al. [112] [26797792]	27 items designed as 5 points Likert scale	Last 2 weeks	1. Symptoms 2. Emotions 3. Social life/functioning 4. Skin	0–108 total points Self-rated QoL impairment cut-off 29 points: mild 50 points: moderate 71 points: severe
Mastocytosis Quality of Life Questionnaire (MQLQ)	Van Anrooij et al. [105] [27089859]	49 items designed as 0 to 6 points scale	No specific evaluation time frame	1. Fatigue and mental health 2. Anaphylaxis 3. Skin symptoms 4. Bone symptoms 5. Un-familiarity 6. Flushing 7. General symptoms 8. Triggers	0–294 total points Cut-off not defined
Mastocytosis Symptom Assessment Form (MSAF)	Van Anrooij et al. [105] [27089859]	20 items designed as 0 to 10 points scale	No specific evaluation time frame	1. Severity of symptoms 2. Impact of fatigue on daily functioning	0–200 total points Cut-off not defined
Mastocytosis Activity Score (MAS)	Siebenhaar et al. [104] [29405310]	9 items designed as 0 to 5 scale	Daily-evaluation of the last week	1. Skin symptoms 2. Gastrointestinal symptoms 3. Others	0–252 total points Self-rated overall disease severity cut-off 11 points: mild 28.1 points: moderate 41.4 points: severe

#### Cutaneous mastocytosis-specific QoL tools

In 2016, two specific QoL questionnaires (MC-QoL and MQLQ) were developed [105, 112]. The MC-QoL explores 4 domains, including symptoms, emotions, social/life functioning, and skin, while the MQLQ focuses upon fatigue and mental health, anaphylaxis, skin symptoms, bone symptoms, and possible triggers (Table 3). Interestingly, although affecting a relatively low percentage of patients compared to other symptoms such as itching or fatigue, recurrent anaphylaxis shows a relevant impact on patients’ QoL with a Mean Importance (MI), defined as the mean of all values given by patients to each item according to the burden it displays on everyday life, of 3.39 according to the MC-QoL (range: 2.20–3.51), and 2.8 according to the MQLQ (range: 0–6) [105, 107, 108, 112, 113]. Moreover, recent studies are investigating the potential relationship between mastocytosis and psychological comorbidities [106, 114]. Vermeiren et al. [106], indeed, asked a total of 50 patients affected by either cutaneous or systemic mastocytosis to answer the questions of two different questionnaires (90-Item Symptom Checklist = SCL-90 and 36 Item Short-Form Health Survey = SF-36) exploring wide-spectrum psychological functions [106]. Surprisingly, according to SCL-90, mastocytosis patients reported lower scores compared to the general population in different categories such as depression, somatization, sleep disorders and inadequacy of acting and thinking, while scoring generally better than people suffering from chronic pain [106]. However, when answering the SF-36 questions, mastocytosis patients appeared to perceive worse body pain than cancer patients, also gaining comparable results regarding vitality and global health status awareness [106]. Interestingly, no differences were found between patients affected by cutaneous forms of mastocytosis and patients affected by systemic ones, thus underling once more how the impact on patients’ QoL does not directly depend on the severity of the disease [106]. However, even if psychological impairment may be a direct consequence of the impact of the disease on the QoL, several Authors believe that depression may be a specific and molecular-based symptom of the disease, related to the constitutional MC activation, which seems to induce the hyperactivation of the indoleamine-2,3-dioxygenase enzyme, thus both enhancing the conversion of tryptophan into kynurenine and reducing its conversion into serotonin [114].

#### Impact of treatment on QoL in patients with cutaneous mastocytosis

In order to address the unmet needs of patients, several drugs, including both traditional ones such as rupatadine and more innovative ones such as omalizumab and midostaurin, have been administered to improve patients’ QoL with encouraging results [115–118]. Rupatadine indeed was proved to be effective in reducing all mastocytosis-related symptoms, improving pruritus and decreasing the need for emergency drugs in 30 patients affected by mastocytosis (23 systemic and 7 cutaneous), when compared to placebo [115]. Moreover, in a study involving 116 patients affected by either cutaneous or systemic mastocytosis, midostaurin has been related to a significant improvement of the SF-12 and Memorial Symptom Assessment Scale scores, with a stable maintenance of the total scores for at least 36 months of follow up [116]. Omalizumab is a therapeutic tool that can be used off-label in patients with recurrent mediator-related symptoms not adequately controlled with the standard of care. Therefore, a recent systematic review of the literature demonstrated that omalizumab can drastically improve the typical symptoms of the disease with a first clinical response after 2.3 months (range: 1–6 months) [117]. However, only one study directly assessed the impact that omalizumab can have on patients’ QoL, thus demonstrating a significant improvement of the Visual Analogue Scale (VAS) score from a basal average score of 8 to a final average score of 2 at last follow up [118]. However, although an increasing interest in mastocytosis patients’ QoL is emerging from the current literature, all studies deal with adult patients with almost no references to the impact of the disease on pediatric patients as well as on their families. Only one study focused indeed upon the impact of UP on the QoL of 37 pediatric patients, reporting episodes of bullying and teasing in 15 patients as well as a general feeling of embarrassment in 12 patients [113]. For these reasons, more studies are needed to focus upon these aspects, also considering that, even if in pediatric patients mastocytosis is usually exclusively cutaneous and indolent, the general QoL does not strictly reflect the severity of the disease [109].

### Quality of life in patients with urticaria

Urticaria is a heterogeneous group of disorders affecting skin and mucosal tissues characterized by the rapid occurrence of wheals, angioedema or both, the latter defining the urticaria-angioedema syndrome. The wheal is a skin lesion presenting with a central edema of variable size, surrounded by erythema, and associated with itching or, more rarely, feeling of warmth, that are transient, with spontaneous resolution in less than 24 h, and with no relics. Classification of urticaria is based on duration of clinical manifestations and on causative agents [119]. Acute urticaria is defined by a duration of symptoms less than 6 weeks, while for chronic urticaria (CU) recurrent appearance of itchy wheals, angioedema, or both lasts more than 6 weeks. It is estimated that 12–22% of the population has suffered at least one subtype of urticaria during life [120], but only a small percentage (estimated at 7.6–16%) has acute urticaria (AU), while the chronic urticaria affects a considerable part of the population worldwide with overall lifetime and point prevalence rates of 1.4% and 0.7%, respectively [120]. The AU has a limited impact on the patient’s life, otherwise the burden of CU for patients, their family and friends, the healthcare system and society is substantial [121]. Previously, O'Donnell et al. showed that health status scores in CU patients are comparable to those reported by patients with coronary artery disease [122]. There are several validated questionnaires helping to better define the burden of disease on patient’s life as the CU quality of life questionnaire (CU-Q2oL), the urticaria activity score (UAS), the urticaria control test (UCT), the angioedema activity score (AAS), the AE-QoL and the DLQI [121, 123–127]. Improving the QoL in patients with CU is of the outmost importance, because this disease has a very great impact on patients’ life, in terms of both duration and comorbidity. Omalizumab improves significantly patients’ QoL, even if larger and standardized prospective studies are needed for better evaluation. Moreover, it should be evaluated the impact on QoL of the new available drugs to have a different therapeutic choice in case antihistamines or omalizumab could not be available or effective.

#### Urticaria-specific QoL tools

UAS, AAS and UCT are indicated to monitor disease control, while the CU-Q2oL, AE-QoL and DLQI are used to evaluate QoL impairment. These questionnaires are very useful, even if they are not always applicable in the daily clinical practice of patients with CU. The available data indicate that urticaria markedly interfered with sleep and daily activities. More than 20% of patients reported ≥ 1 h per week of missed work and the productivity impairment was 27%. These effects increased with increasing disease activity. Furthermore, patients are concerned about the disabling effects and the stigma related to the appearance of angioedema or urticaria and this increases the burden of the disease. Therefore, the efficacy of CU treatment has a significant impact on patients’ QoL and, consequently, on healthcare resources and costs [128].

#### Impact of treatment on QoL in patients with urticaria

The treatment of CU is characterized by a stepwise approach in which second-generation H1-antihistamines are the first-line medication for the initial management, according to the updated version of the EAACI/GA^2^LEN/EDF/WAO guidelines [86]. Second-generation H1-antihistamines have been preferred over the first-generation antihistamines for their better safety and efficacy profile, and their less sedative adverse effects. Continuous administration of antihistamines at standard dose is required, increasing up to four times the licensed dose (off-label use), as second line treatment, if symptoms are not adequately controlled after 2 to 4 weeks or earlier if the symptoms are intolerable. The major inconveniences for patients suffering from CU is firstly, the long duration of the disease (around two to five years) which therefore requires prolonged therapies with antihistamines, and secondly, the fact that the treatment’s adherence tends to decrease over time because some patients discontinue medications when CU is asymptomatic [129, 130]. Moreover, most patients do not respond to the standard antihistamine dose, and they need an up-dosing, as reported in a recent observational study where 82% of patients did not respond to standard-dosed against 26% of non-responding if higher doses of antihistamines were administered [131]. A systematic review showed that up dosing antihistamines significantly improves control of itching, but not weal number [132]. Unfortunately, some patients refuse to increase antihistamine dosage especially when it is higher than licensed dosage, because they are afraid of potential side effects, such as drowsiness, which can further contribute to a negative impact on QoL. More recently, efficacy and safety of up dosing of second generation H1-antihistamines has been reported. Most of the recent literature is focused on evaluation of bilastine, which showed a high safety profile with no interference with performance and learning abilities in both adults and children (age ≥ 6 years old) improving QoL of patients suffering from urticaria [133]. A multicenter, randomized, double-blind, placebo-controlled study in Japanese population with CU showed the efficacy of bilastine compared to placebo, maintained up to 52 weeks in an open-label study [134, 135]. Several studies have evaluated possible interactions of bilastine with daily activities and its influence on QoL. Demonte et al. evaluated the effect of 7 days treatment with 20 mg daily bilastine on the driving ability rolling out possible interaction [136]. In a comparative study on a cohort of 58 adult patient affected by CU, bilastine 20 mg daily was found to be less-sedative and more effective long-term treatment compared to levocetirizine 5 mg daily in term of UAS7 reduction at the end of treatment (UAS score mean change of −31.6 ± 1.5 during bilastine 20 mg therapy compared to mean change of −27.4 ± 1.7 during levocetirizine 5 mg daily), but with similar effect on improvement of QoL and urticarial-induced global discomfort in a follow-up period of 42 days. Both drugs statistically improved patients’ QoL with comparable results: 94,8% of patients reported large to very large effect of chronic spontaneous urticaria (CSU) on QoL (DLQI score 11–30) in contrast to 8.6% of patient who referred small to moderate impairment of their QoL (DLQI 0–10) at the end of the study. In addition, long-term administration of levocetirizine 5 mg daily was associated with greater onset of somnolence compared to bilastine 20 mg daily (63% Vs 12.9%) [137]. Interestingly, significantly clinical improvement with bilastine compared to levocetirizine was observed only after 6 weeks of treatment (42 days) while it was comparable after 14 and 28 days in accordance with the findings of Zuberbier et al. reporting after 28 days of treatment changes in DLQI and UAS scores [138]. Omalizumab is the first non-antihistamine drug licensed for the treatment of CSU, as third line treatment, according to international guidelines [86]. Approved for the treatment of antihistamine-resistant CSU in patients 12 years or older (300 mg subcutaneous every 4 weeks), omalizumab has been proved to be safe and effective in several randomized controlled trials [139, 140] and several real-world studies [141, 142], as well as to improve QoL in CSU patients [143]. There are many questionnaires to evaluate the effect of omalizumab on quality of life, but the most utilized in registration studies was DLQI. In the studies ASTERIA I, ASTERIA II and GLACIAL omalizumab 300 mg every 4 weeks significantly improved the total DLQI scores compared to placebo (*p* < 0.01; all studies) [144]. According to a recent EAACI’s review on the effectiveness and safety of omalizumab, the normal dosage of 300 mg q4w improved QoL reducing at the same time the DLQI in 6 RCTs and the CU-Q2ol in 3 RCTs compared to standard of care [145]. The clinical benefits associated with omalizumab therapy in real-world management of CSU are less known. A meta-analysis of 67 studies, assessing the real-world effectiveness of omalizumab in CSU (with or without angioedema), showed a significant QoL improvement in patients treated with omalizumab with a reduction of the DLQI score (13.9 points) in 6 studies involving 84 patients and a reduction of CU-Q2oL (42.3 points) in 3 studies involving 70 patients [146]. In a systematic review of 84 publications, Bernstein et al. reported the real-world effectiveness of omalizumab for treatment of CSU. QoL outcomes improved by 76.2% (DLQI; 6 studies and 70 patients) and 71.1% (CU-Q2oL; 5 studies and 72 patients) [147]. There is an increasing number of real-world studies that evaluate the effectiveness of the up dosing of omalizumab in CSU (450 mg o 600 mg q4w), proving improvements in UAS7, UCT, and quality of life scores in patients who were not responding sufficiently to standard dose of omalizumab [148]. Some RCTs proved the efficacy of omalizumab in CSU patients with angioedema, associated symptom in 33–67% of patients [149], resulting in an improvement of the frequency and the severity of the angioedema itself (ASS) and the QoL (AEQoL) [150, 151]. In the RCTs X-ACT, that included only CSU patients who have had 4 or more angioedema episodes at timeline 0, treatment with omalizumab 300 mg q4w for 28 weeks significantly decreased the AAS and AE-QoL, with evident improvements already in the first 4 weeks [152]. Maurer et al. showed that patients treated with omalizumab 300 mg had more clinical benefits, in terms of QoL improvement and reduction of the frequency of the angioedema episodes, as compared to patients treated with omalizumab 150 mg or 75 mg [153]. To date, no RCT has included CSU patients that suffer from recurrent angioedema without wheals. Some case reports document an improvement of ASS and AE-QoL in individuals after omalizumab treatment [154]. Ciclosporin is an off-label drug for urticarial recommended by 2021 international guideline as four-line treatment in patients not responding to omalizumab in combination with antihistamines treatment [86]. It directly inhibits mast cell degranulation in a moderate way as well as targeting T cells involved in pathogenetic mechanisms of urticaria. Although effective, its use is limited by several potential adverse effects such as hypertension, nephrotoxicity, headache, nausea, and gastrointestinal problems [155–157]. A close monitoring of blood pressure and renal function is needed in patients who are taking ciclosporin for CU resulting in considerable discomfort for patients that further impaired their QoL, even if the longer the duration of use, the higher the risk of side effects. There are few studies regarding ciclosporin and QoL in patients with urticaria. Two parallel multicenter retrospective observational studies [158] have compared outcomes and change of DLQI in patients treated with omalizumab or cyclosporin highlighting a greater improvement in mean DLQI in patient treated with omalizumab than ciclosporin (75% reduction in DLQI was achieved in 79% and 41%, respectively; while 90% reduction in DLQI was achieved in 65 and 18%, respectively). As bias of the study, DLQI was subjected only to a minority of patients examined. Clinical outcomes of patients treated with ciclosporin (dosage 3–4 mg/kg/die) were not evaluated through UAS7 score and clinical remission was achieved only in 17% (vs omalizumab treatment 42%), clinical improvement in 55% while no response in 28% of patients. Several novel treatments for CU are currently under development [159]. Ligelizumab is a high affinity humanized monoclonal anti-IgE antibody. In the phase 2b dose-finding trial, a superior efficacy and persistent response, in terms of both UAS7 and AAS scores, was observed in patients treated with ligelizumab compared with the omalizumab group [160]. Other biologics, already approved for the treatment of AD and/or asthma and/or chronic rhinosinusitis with nasal polyposis, could be off-label used in CU. Lee and Simpson evaluate UAS at baseline and 3 months after dupilumab in 6 patients affected by both AD and CU, unresponsive to omalizumab, with good results [161]. There are two ongoing RCTs to estimate efficacy and safety of dupilumab in CU. Several case reports of mepolizumab, reslizumab and benralizumab, used to treat CU occurred in asthma patients, suggest that blocking of IL-5 axis could be a promising option [159]. Maurer et al. described a sudden improvement of symptoms and QoL of a patient with CU and cold urticaria with UCT score of 16 four weeks after the first injection of reslizumab, indicating complete disease control. This score was maintained for at least four months after treatment [162]. There is no further available data about QoL for these treatments yet.

## Conclusions

Allergic and immunologic skin diseases have a significant impact on patients' QoL. Physicians diagnose diseases to care for and cure patients. The assessment of QoL is important for clinical decision and relies on questionnaires which are answered directly by the patient, thus QoL is indeed in the eye of the beholder. As Sir William Osler stated in *Aequinimitas* more than a century ago, the value of experience is in seeing wisely, in this case, balancing efforts in treating disease with the burden of the illness in a patient’s life.

## Data Availability

Not applicable.

## Abbreviations

AAS: Angioedema activity score
ACD: Allergic contact dermatitis
AD: Atopic dermatitis
ADvSM: Advanced Systemic Mastocytosis
AE: Angioedema
AE-QoL: Angioedema quality of life questionnaire
AU: Acute urticaria
CADIS: Childhood Atopic Dermatitis Impact Scale
CD: Contact dermatitis
CDLQI: Children’s Dermatology Life Quality Index
CM: Cutaneous mastocytosis
CU: Chronic urticaria
CU-Q2oL: CU quality of life questionnaire
C1-INH: C1 esterase inhibitor
DLQI: Dermatology Life Quality Index
DSQL: Dermatology-Specific Quality of Life
EQ-5D: EuroQol-5D
EQ VAS: EuroQol visual analogue scale
FQLI: Fragrance Quality of Life Index
HAE: Hereditary angioedema
HAEA-QoL: The United States HAE Association QoL questionnaire
HAE-QoL: Hereditary Angioedema QoL questionnaire
HRQoL: Health Related Quality of Life
ICD: Irritant contact dermatitis
ISM: Indolent Systemic Mastocytosis
MAS: Mastocytosis activity score
MC: Mast cells
MCID: Minimal clinically important difference
MC-QoL: MastoCytosis Quality of Life Questionnaire
MI: Mean importance
MQLQ: Mastocytosis Quality of Life Questionnaire
MSAF: Mastocytosis Symptoms Assessment Form
NHP: Nottingham Health Profile
NRS: Numerical Rating Scale
PUVA: Psoralen plus UVA
QoL: Quality of life
QOLHEQ: Quality of life in Hand Eczema Questionnaire
QoLIAD: QoL Index for Atopic Dermatitis
SCL-90: 90-Item Symptom Checklist
SF-12: Short Form 12 Health Survey
SF-36: Short Form 36 Health Survey
SIAAIC: Italian Society of Allergology, Asthma and Clinical Immunology
SIDAPA: Italian Society of Allergological, Occupational and Enviromental Dermatology
SM: Systemic mastocytosis
UAS: Urticaria activity score
UCT: Urticaria control test
WPAI: Work Productivity and Activity Impairment
